# Short-Term Effects of High-Frequency Chest Compression and Positive Expiratory Pressure in Patients With Cystic Fibrosis

**DOI:** 10.4021/jocmr697w

**Published:** 2011-11-10

**Authors:** Valentina Fainardi, Francesco Longo, Silvia Faverzani, Maria Candida Tripodi, Alfredo Chetta, Giovanna Pisi

**Affiliations:** a Cystic Fibrosis Unit, Departmet of Pediatrics, University Hospital, Parma, Italy; b Lung Function Unit, Department of Cardio-Pulmonary Diseases, University Hospital, Parma, Italy

## Abstract

**Background:**

Cystic fibrosis patients require daily airway clearance therapies. The primary objective of this study was to compare the short-term efficacy of high-frequency chest compression and positive expiratory pressure mask on expectorated sputum, pulmonary function, and oxygen saturation in patients with CF hospitalized for an acute pulmonary exacerbation.

**Methods:**

A controlled randomized cross-over trial with 24 hours between treatments was used. Thirty-four CF patients (26 ± 6.5 years) were included in the study. Before and 30 minutes after each treatment were recorded: pulmonary function testing, oxygen saturation, and perceived dyspnea. Preference for the two devices was assessed.

**Results:**

No statistically significant difference between high-frequency chest compression and positive expiratory pressure mask was found in sputum production and in lung function testing. A reduction in SpO_2_ was found after positive expiratory pressure mask (98 ± 1.0% versus 97 ± 1.2%; P < 0.001). Both treatments induced a statistically significant increase in Borg scale for dyspnea without differences between them. Patients reported greater satisfaction with positive expiratory pressure mask than with high-frequency chest compression (P < 0.001).

**Conclusion:**

High-frequency chest compression and positive expiratory pressure mask have comparable short-term effects on expectorated sputum and lung function. Although positive expiratory pressure mask was associated with a lower SpO_2_, it was better tolerated than high-frequency chest compression.

**Keywords:**

Airway clearance therapies; High-frequency chest compression; Sputum; Cystic fibrosis

## Introduction

In cystic fibrosis (CF) mucociliary clearance is impaired, and infected airway secretions gradually cause lung tissue destruction. As a result, peripheral airways tend to collapse leading to trapping air, mucus obstruction, and chronic pulmonary infections [1]. Airway clearance therapies (ACTs) are one of the cornerstones of CF treatment. Main goal of the ACTs is to improve ventilation and enhance airway clearance through the mobilization and expectoration of mucus. Over the last years new ACTs has been developed in the attempt to provide more independence, better quality of life and increased compliance through a more comfortable and less time consuming technique [2].

High-frequency chest compression (HFCC), an oscillatory device, is a self-administered chest physiotherapy that with an inflatable compressive jacket generates airways vibrations, increases airflow, and mobilizes mucus. Though HFCC was firstly used in 1990 showing greater results than conventional physiotherapy in mucus clearance [3], literature still shows conflicting results about the effectiveness of this therapy.

Compared with conventional physiotherapy (i.e. postural drainage and percussion, PD&P), Kluft et al reported that HFCC produced a greater amount of sputum over a six-day period [4], and Warwick first demonstrated a positive long-term effect of HFCC on FEV_1_ (forced expiratory volume in 1 second) and FVC (forced vital capacity) [5]. Other studies found no difference in pulmonary function [6-8], sputum production [6,7,9], and in oxygen saturation during or following treatments [8]. If compared with active cycle of breathing techniques, HFCC resulted less effective in improving lung volumes and mucus expectoration [10]

Patients preference, a critical determinant of ACTs adherence [11], was discussed by Braggion and Varekojis, without statistical evidence [6,9], and by Arens who reported greater satisfaction with HFCC [8]. The latest study comparing HFCC, postural drainage and Flutter device, although early interrupted for high dropout rate, showed that patients were more satisfied with oscillating devices [12].

A recent review concluded that oscillating devices are not superior, in terms of effectiveness and acceptability, to any other forms of physiotherapy techniques [13].

A more common airway clearance device is the positive expiratory pressure (PEP) mask that, increasing air flow to small peripheral airways, is thought to have beneficial effects on sputum production [14]. To date, in CF patients HFCC was compared to PEP in only three trials [6,15,16]. Two of them found no statistical difference in sputum production [6] and lung function with both devices [6,16]; the more recent trial by Osman et al reported a statistically significant greater volume of expectoration by usual ACTs but no difference in lung volumes [15]. Notably, the results from the latter study came from a comparison between HFCC and usual ACTs including, not only PEP, but also active cycle of breathing techniques with postural drainage and percussion, autogenic drainage, and Flutter device.

The aim of the present study was to compare the short-term efficacy, assessed by sputum production, of HFCC and PEP in hospitalized adult patients with mild to moderate CF lung disease. Secondary outcomes included pulmonary function tests, and oxygen saturation before and after each airway clearance treatment, adverse effects and patient preference.

## Materials and Methods

### Subjects

Participants were current patients of the Cystic Fibrosis Unit of Parma University Hospital, admitted to ward for management of an acute exacerbation of respiratory symptoms. The inclusion criteria were a confirmed diagnosis of CF, age > 18 years, mild to moderate lung function impairment (FEV_1_ > 60%), chronic infection by *Pseudomonas aeruginosa*, and an infective pulmonary exacerbation as defined by the following symptoms: increased cough, volume and purulence of sputum, fatigue, dyspnoea, decrease in pulmonary function, and weigh loss [17]. The patients were treated with intravenous antibiotics (combination of β-lattamic plus aminoglycoside) for at least 15 days. In addition, routine medication was continued throughout the study.

Exclusion criteria were patients on steroid therapy, patients awaiting for lung transplant, affected by concomitant malignancies, haemoptysis, and rib fractures, or pregnancy. Informed consent was obtained from all individuals and the study was approved by the Ethical Committee of the University of Parma.

Enrolled patients routinely used PEP mask therapy three times daily. Before starting the study each patient was familiarized with HFCC through a certified respiratory therapist who explained how the device operated.

### Study design

A randomized cross-over trial with 24 hours between treatments was used to compare HFCC and PEP mask. Patients were admitted on day 0 and, after clinical and functional evaluation, underwent either PEP mask therapy on day 1 and HFCC treatment on day 2 or vice-versa. Subjects were assigned to one of the two treatments by numbering them consecutively: odd-numbered subjects were assigned to perform HFCC on day 1 and PEP therapy on day 2; even-numbered subjects did the opposite. All study sections were at morning, between 8.00 h a.m. and 10.00 h a.m.; each airway clearance treatment session lasted 30 minutes and was assisted by the same physiotherapist.

On each study section, immediately before and 30 minutes after each treatment, pulmonary function testing, transcutaneous pulsed arterial oxygen saturation (SpO_2_, %), and perceived dyspnea ratings were recorded. During each treatment session and over 30 minutes the amount (mL) of sputum was collected and measured with a burette. An independent observer, blind to the method of airway clearance used, performed the spirometry and weighed the sputum. At the end of the trial patients were asked if they felt comfortable with the two devices answering to a Yes/No question.

### High-frequency chest compression (HFCC)

HFCC is a portable mechanical method of self-administered chest physiotherapy. HFCC was delivered by a pneumatic vest (The Vest Airway Clearance System Model 4 by Hill-Rom®, St. Paul, MN, US), appropriately sized, that surrounded the thorax of the patient. Vest was connected by two tubes to the air-pulse generator and inflated by a constant positive pressure with a 15-20 Hz frequency of air pressure oscillations. The pulse pressure (ranging 6-10 on the 1-10 Vest’s scale) was set according to individual patient’s reported comfort. Subjects remained in the upright sitting position throughout the 30 minutes treatment session. During the HFCC patients were invited to huff and to cough actively (ranging 3-5 times) as to expectorate dislodged bronchial secretions.

### Positive expiratory pressure (PEP)

PEP mask (Astra Tech AB, Molndal, Sweden) has a one-way valve to which an expiratory orifice resistor is attached. When the patient exhales trough the resistor a positive pressure is generated in the airways (10-20 cm H_2_O) and collapsed lung are re-inflated raising the functional residual capacity. According to the guidelines, the patients, placed in a sitting position, were instructed to hold tightly the mask over the mouth and the nose and were invited to breath using slightly active breaths for 30 minutes. Each treatment consisted of 15 breaths approximately followed by more cycles of forced expiration (huff). The number of cycles within a treatment session was adapted to individual comfort [18].

### Pulmonary function testing and perceived dyspnoea ratings

Spirometry was performed with a computerized spirometer (VMAX22 PFT Sensormedics, Yorba Linda, CA, US) according to the international standards [19]. FEV_1_ (%), forced expiratory flow 25-75 (FEF_25-75_, %), and FVC were recorded and expressed as percent of predicted value. SpO_2_ was measured by a fingertip pulse oximeter (Nellcor P200, Puritan Bennett LLC, Boulder, CO, US).

Perceived dyspnoea ratings were obtained by using a modified Borg scale labeled from 0 (no symptoms) to 10 (maximum bearable) [20].

### Statistical analysis

Data were expressed as mean ± SD or percentage, as appropriate. Paired t-test or Chi Square analysis was used for comparisons, when appropriate. A P value < 0.05 was considered as statistically significant. All analyses were conducted using SPSS Statistics 16.0 (IBM).

## Results

Thirty-six CF patients were enrolled. Thirty four of 36 (20 females) completed the study, two patients withdrew from the study because of discomfort with HFCC device. In patients who completed the study, both PEP and HFCC techniques have not been associated with statistically significant side effects. Demographics and baseline characteristics of the 34 patients are shown in Table 1.

**Table 1:** Demographics of Enrolled Participants

**Demographics and baseline characteristics**	**value**
Age (year)	26 ± 6.5
Gender (M / F)	14 / 20
BMI (kg/m^2^)	19.72 ± 6.35
SpO_2_ (%)	97.7 ± 1.4
FEV_1_ (% pred)	67 ± 17

BMI: body mass index; SpO_2_: transcutaneous pulsed arterial oxygen saturation; FEV_1_: forced expiratory volume in 1 second. Data are presented as mean ± SD or ratio as appropriate.

FEV_1_, FEF_25-75_ and FVC measured before and after PEP mask and HFCC treatment session are reported in Table 2. No statistically significant difference was found after both treatments. As compared to baseline values, small but statistically significant decrease in SpO_2_ values was found after PEP treatment (98 ± 1.0% versus 97 ± 1.2%; P < 0.001), but not after HFCC treatment (97 ± 1.6% versus 97 ± 1.2%) (Table 2).

**Table 2:** Lung Function Values: Before (Pre) and After (Post) PEP and HFCC Treatments

**Device**	**FEV_1_**		**FEF_25-75_ (%)**		**FVC (%)**		**SpO_2_ (%)**	
	**(% predicted)**							
	pre	post	pre	post	pre	post	pre	post
HFCC	67 ± 17	66 ± 17	34 ± 21	33 ± 21	88 ± 17	87 ± 16	97 ± 1.6	97 ± 1.2
PEP	67 ± 16	67 ± 16	34 ± 20	34 ± 19	88 ± 15	87 ± 15	98 ± 1.0	97 ± 1.2*

FEV_1_: forced expiratory volume in 1 second; FEF_25-75_: forced expiratory flow 25 - 75%; FVC, forced vital capacity. *P < 0.001. No statistically significant difference was found in spirometric values before and after both treatments. A statistically significant decrease in SpO_2_ values was found after PEP treatment.

**Figure 1:**
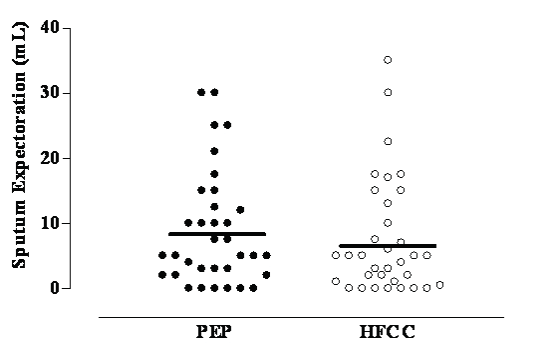
Mean and individual values of sputum expectoration after PEP and HFCC in 34 CF patients.

The average amount of sputum expectorated after PEP (8.8 ± 8.8 mL) and after HFCC (7.5 ± 8.9 mL) did not significantly differ (Fig. 1). Both treatments induced a statistically significant increase in Borg scale for dyspnea without differences between them (Table 3).

**Table 3:** Borg Dyspnea Scale Before (Pre) and After (Post) PEP and HFCC Treatments

****	**Pre**	**Post**	**P value**
HFCC	0.7 ± 0.9	1.5 ± 1.7	< 0.01
PEP	0.9 ± 1.2	1.6 ± 1.6	< 0.001

Both treatments induced a statistically significant increase in Borg scale for dyspnea, without differences between them.

Forty-one per cent of the patients expressed no preference for the devices reporting that they found comfortable both techniques, 50% preferred PEP therapy, and 9% HFCC (P < 0.001).

## Discussion

The present study showed that in CF patients, hospitalized for pulmonary exacerbation, both PEP and HFCC had comparable short-term effect on sputum production and lung function. A small but statistically significant reduction in oxygen saturation was found following PEP therapy; but not after HFCC. PEP therapy was much preferred than HFCC.

Our study confirmed the findings by previous reports that found no statistically significant differences between baseline and after intervention values with either HFCC or PEP, both in sputum production and pulmonary function measures [6,7]. By contrast, Kluft et al reported a greater effect of HFCC on sputum production compared to other conventional techniques [4]. However, it is of note that in that study a nebulised saline was administered throughout the treatment to prevent airway drying. Taken together these findings suggest that the nebulised saline solution could magnify the effect of HFCC. The absence of a statistically significant modification in lung volumes suggests that spirometry may be inadequate to detect the short-term effects of ACTs.

We found a small but statistically significant reduction in oxygen saturation when patients underwent PEP therapy. But the results showed that this finding was not due to a greater mucus mobilization. An opposite result was reported in the study by Darbee et al, where oxygen saturation increased during PEP and decreased during HFCC. These modifications were not sustained after treatment [16]. The discrepancy between our and Darbee’s results can be explained by differences in the study protocol. In our study, oxygen saturation was recorded 30 minutes after therapy, while in the study by Darbee et al it was recorded during therapy [16]. Importantly, ours and Darbee’s results may suggest to monitor oxygen saturation during and after airway clearance treatments, mainly in CF patients with severe lung disease where SpO_2_ desaturation may be greater and more clinically significant.

A recognized positive feature of high-frequency chest wall oscillations is that it is self-administered and do not require the patient to change positions [4]; accordingly, HFCC might preserve independence and it may be useful in fatigue patients who cannot tolerate additional respiratory work. However, in spite of that and by contrast with previous studies, reporting greater satisfaction and compliance with oscillatory devices compared to conventional chest physiotherapy [8,12], our patients expressed more satisfaction with PEP therapy than HFCC. Furthermore, two out of 36 patients withdrew from the study because of discomfort with HFCC device. It is conceivable that the preference for HFCC demonstrated by the previous studies may have been influenced by the study protocol modalities. Notably, in these studies physiotherapy was self-managed, performed at home, and the compliance to the treatment was not constantly assured by a physiotherapist.

We are aware of the limitations of our study. Firstly, we assessed the short-term effect of the two airway clearance devices and we cannot draw any conclusion about their long-term effects. To date, few trials have compared the potential benefits of HFCC with other forms of airway clearance and the studies were mostly 1 or 2 days long. Recently, Sontag et al [12] started a 3 years-longitudinal trial to compare PD&D, HFCC and Flutter device but they ended early the study because of the high rate of withdrawal. Secondly, in the present study we considered fresh sputum, as outcome measure. Scherer et al [7] reported that evaluating ACTs efficacy through fresh sputum weight might be inaccurate because of the contaminations of lower airways secretions with saliva, due to its high water content. However, no difference has been found between dried and fresh sputum, when considered as outcome measures to assess the efficacy of different airway clearance devices [4].

In conclusion, our study showed that HFCC was comparable to PEP in terms of sputum production and lung function effects, but not in terms of acceptability. To date no studies have clearly demonstrated the optimal airway clearance, likely due to different patients features, such as age, compliance, concomitant disease, and severity of lung involvement by CF [21]. Thus, airway clearance treatment should be individualized, and its efficacy periodically reassessed evaluating patient preference and objective measures, such as lung function test or exacerbation rate.
